# Orthodontists’ Perceptions, Attitudes, and Adoption of Artificial Intelligence: A Scoping Review

**DOI:** 10.3390/dj14050257

**Published:** 2026-04-28

**Authors:** Salvatore La Rosa, Ludovica Nucci, Cristina Grippaudo, Rosalia Leonardi, Alessandro Polizzi

**Affiliations:** 1 Department of General Surgery and Medical-Surgical Specialties, Section of Orthodontics, University of Catania, Policlinico Universitario “Gaspare Rodolico—San Marco”, Via Santa Sofia 78, 95123 Catania, Italy; salvo.larosa11@live.it (S.L.R.); rleonard@unict.it (R.L.); 2 Department of Mental and Physical Health and Preventive Medicine, University of Campania Luigi Vanvitelli, 80138 Naples, Italy; ludovica.nucci@unicampania.it; 3 Complex Operative Unit of Dental Clinic (UOC), Department of Neurosciences, Sensory Organs and Thorax, Fondazione Policlinico Universitario A. Gemelli IRCCS, 00168 Rome, Italy; cristina.grippaudo@unicatt.it; 4 Department of Head, Neck and Sensory Organs, Università Cattolica del Sacro Cuore, 00168 Rome, Italy

**Keywords:** orthodontics, artificial intelligence, AI perception

## Abstract

**Background:** Artificial intelligence (AI) is increasingly utilized in orthodontics for diagnosis, treatment planning, and clinical management. Nevertheless, considerable variation persists in orthodontists’ knowledge, attitudes, utilization patterns, recommendations, and concerns regarding AI. The aim of this scoping review was to synthesize survey-based evidence on orthodontists’ perceptions and experiences with artificial intelligence. **Methods:** A systematic literature search was performed in PubMed, Embase, Scopus, and Web of Science to identify survey-based studies evaluating AI awareness, applications, attitudes, recommendations, and concerns among orthodontists, residents, postgraduate students, and academicians. **Results**: Seven studies involving a total of 1772 participants were included. Overall, postgraduate students and practicing clinicians demonstrated relatively limited knowledge of AI, whereas academicians exhibited a higher level of awareness. Although routine clinical implementation of AI remains limited, it was most frequently applied—or perceived as beneficial—in cephalometric and cone beam computed tomography (CBCT) analysis, orthognathic surgery planning, and treatment outcome prediction. The majority of participants supported the promotion of AI and the integration of AI education into orthodontic curricula. However, concerns persisted regarding insufficient technical expertise, high costs, ethical and legal challenges, reduced patient engagement, and the potential for diagnostic or procedural errors. Consistent variations related to age and professional role were observed across studies among academicians, postgraduate students, orthodontists, and residents. **Conclusions:** This scoping review seems to suggest a growing interest in and generally positive attitudes toward AI among orthodontists and trainees. However, the evidence base is limited to a small number of studies, heterogeneous and predominantly based on cross-sectional survey data. For these reasons, findings should be interpreted cautiously. Variability in knowledge and use persists, and integration into practice remains inconsistent. Further research is needed to support effective and evidence-based implementation.

## 1. Introduction

Artificial intelligence (AI) comprises a set of computational technologies designed to simulate or augment human cognitive functions, including reasoning, learning, planning, and pattern recognition [1]. Drawing on computer science, statistics, neuroscience, and engineering, AI systems analyze large-scale datasets through mathematical modelling and algorithm training to generate autonomous or semi-autonomous predictions and decisions.

Key AI subfields relevant to medicine and dentistry include Machine Learning (ML), which enables data-driven performance improvement without explicit programming; Deep Learning (DL), which employs multilayer neural networks to process complex unstructured data such as radiographic images; Natural Language Processing (NLP), which facilitates the extraction and interpretation of information from medical texts and electronic health records; and Big Data analytics, which integrates heterogeneous datasets to support personalized clinical decision-making [2,3,4]. Advances in computational power have accelerated the implementation of increasingly sophisticated algorithms, enhancing diagnostic accuracy, risk stratification, and clinical decision support while reducing interindividual variability and cognitive burden among healthcare professionals [5].

In dentistry and orthodontics, AI applications range from administrative functions to diagnostic analysis, treatment planning, prognostic assessment, radiographic interpretation, automated image analysis, patient monitoring, workflow optimization, and computer-aided design and manufacturing of dental restorations. These technologies aim to improve efficiency, reduce costs, and minimize decision-making errors [6,7,8,9].

Orthodontists’ perceptions and attitudes toward AI play a critical role in its clinical adoption [10,11,12]. Perception involves cognitive recognition and interpretation, whereas attitude reflects a behavioural predisposition toward implementation [13]. Positive professional attitudes and trust in AI facilitate integration into practice, promote patient acceptance, and encourage innovation [8,14,15].

This review examines orthodontists’ knowledge, concerns, and attitudes regarding AI, identifies perceived limitations of current technologies, and highlights future research directions to support effective integration into orthodontic practice.

## 2. Materials and Methods

A scoping review methodology was selected due to the limited number of available studies and the heterogeneity in study designs, populations, and outcome measures. This approach enables the systematic mapping of existing evidence and the identification of knowledge gaps concerning orthodontists’ perceptions of artificial intelligence (AI).

The present review was conducted in accordance with the Joanna Briggs Institute (JBI) methodological framework for scoping reviews and the Preferred Reporting Items for Systematic Reviews and Meta-Analyses extension for Scoping Reviews (PRISMA-ScR) guidelines [16,17] (PRISMA 2020 ScR checklist). The review protocol was prospectively registered in the Open Science Framework (OSF) database: https://doi.org/10.17605/OSF.IO/MVBN6 (accessed on 20 January 2026).

### 2.1. Research Question

A primary research question was formulated to guide the literature search and data extraction process:

“*What are orthodontists’ perceptions of artificial intelligence, including their knowledge, attitudes, concerns, and perceived opportunities for improvement in current AI-based technologies?*”

This question was designed to provide readers with a comprehensive reference framework for understanding the current state of evidence.

### 2.2. Justification

Although the application of AI in orthodontics has been increasingly investigated, comparatively limited attention has been devoted to orthodontists’ perspectives—both among specialists and trainees—regarding this emerging technology. Clinicians’ perceptions are critical, as their acceptance, trust, and preparedness directly influence the successful implementation of AI in clinical practice. To date, no comprehensive review has systematically synthesized and compared existing studies to provide an overarching evaluation of orthodontists’ perceptions of AI, thereby revealing a significant gap in the current literature.

### 2.3. Eligibility Criteria

This review included original cross-sectional studies investigating orthodontists’ knowledge, perceptions, attitudes, and concerns related to AI. The following were excluded: incomplete manuscripts, systematic reviews, narrative reviews, case series, consensus conference reports, and articles published in languages other than English. This selection strategy was adopted to ensure methodological rigor and relevance.

Studies were selected according to the Population–Concept–Context (PCC) framework. The population comprised orthodontists and orthodontic trainees; the concept encompassed perceptions, knowledge, attitudes, and concerns regarding AI; and the context included orthodontic clinical practice and educational environments.

### 2.4. Literature Sources and Search Strategy

Database searches were conducted through January 2026 to identify relevant literature. The search strategy was developed in collaboration with a health sciences librarian and incorporated controlled vocabulary terms and free-text keywords. The following electronic databases were searched: Web of Science, Embase, Scopus, and PubMed. Additionally, the reference lists of included studies were screened to ensure comprehensive source identification. Table 1 presents the detailed search strategies applied to each database.

### 2.5. Data Management

#### 2.5.1. Study Selection

All citations retrieved from the electronic databases were imported into EndNote version 20.6 (Clarivate™, London, UK) for reference management. Duplicate records were removed, and when multiple publications reported preliminary or updated findings from the same study, they were considered only once.

Two reviewers (S.L.R. and A.P.) independently screened the titles and abstracts of all identified records. Full texts were subsequently assessed for eligibility when deemed potentially relevant. Only studies explicitly investigating the perceptions, attitudes, or knowledge of orthodontists or orthodontic students regarding the use of AI were included, while all other articles not directly addressing this topic were excluded. The studies, including orthodontists and other professionals, were extracted for completeness, but only orthodontists’ responses were considered in the synthesis of the findings or in the discussion. The other studies that did not include orthodontists were excluded from the review to maintain consistency with the stated eligibility criteria. Disagreements regarding study inclusion were resolved through discussion with a third reviewer (R.L.) to ensure impartial evaluation. Inter-reviewer agreement was calculated using Cohen’s kappa statistic (k = 0.87).

The screening process yielded a progressive reduction in the number of records, with studies excluded at the title and abstract stage primarily due to lack of relevance to orthodontics or absence of focus on clinicians’ perspectives. Additional exclusions at the full-text stage were based on inadequate population, study design, or insufficient reporting of outcomes of interest. The final selection comprised a subset of studies meeting all inclusion criteria, which were then included in the qualitative synthesis.

#### 2.5.2. Data Extraction

Two reviewers (S.L.R. and A.P.) independently extracted relevant information from each eligible article, with discrepancies resolved through discussion and, when necessary, consultation with a third reviewer (R.L.). The level of agreement between the two reviewers was assessed using Cohen’s kappa statistic (k = 0.87). Extracted data included study characteristics (author, year, country), study design, sample size and population (with particular attention to orthodontists and orthodontic trainees), and key outcomes related to perceptions, attitudes, and knowledge of AI. In studies involving mixed professional groups, only data specifically referring to orthodontists were considered for the synthesis, in line with the predefined eligibility criteria. Additional variables, such as survey methodology, type of questions (closed-ended or open-ended), and main findings, were also recorded to support qualitative comparison across studies.

### 2.6. Data Synthesis

The synthesis of findings followed established methodological frameworks described in previous studies [18]. Extracted data were systematically categorized and organized into thematic domains to enhance clinical relevance (AI awareness, attitudes toward AI, AI Recommendation, current usage of AI and concerns about AI). These domains were designed to comprehensively encompass all pertinent information derived from the included studies.

By building upon methodological approaches adopted in prior research, this strategy ensured a structured, transparent, and comprehensive presentation of the findings.

## 3. Results

### 3.1. Identified Studies

**Figure 1:**
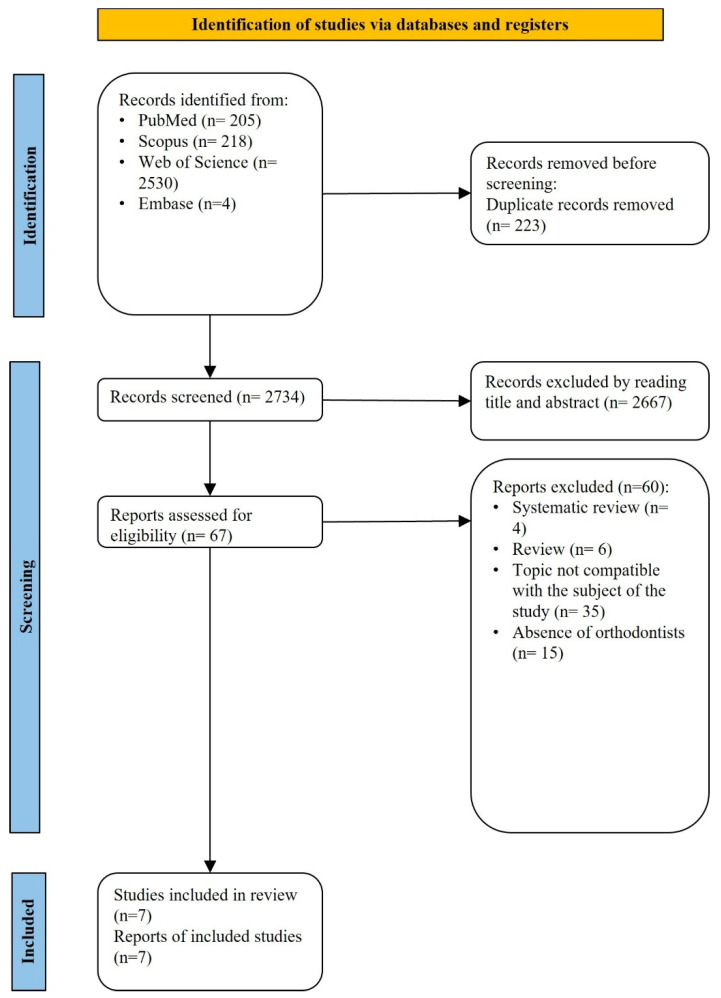
PRISMA flow-chart of the study selection.

The search identified 2957 research articles on the attitudes, knowledge, and views of orthodontists and orthodontic students about AI and its uses in orthodontics from the aforementioned databases. After the removal of duplicate records, 2734 records were identified. Title and abstract screening resulted in the exclusion of irrelevant records, and 67 full-text articles were assessed for eligibility. Following full-text evaluation, 7 studies met the inclusion criteria and were included in the scoping review. A summary of all the data related to study selection is shown in Figure 1.

### 3.2. Summary of Identified Studies

The studies included in the review were cross-sectional surveys carried out in India [12,15,19,20], China [21], Iraq [22] and North America [23], suggesting that Asian nations have been the main focus of this topic. Additionally, the studies looked at orthodontic students’ and orthodontists’ attitudes, knowledge, and perceptions of AI; the results were presented as percentages of responses about basic AI knowledge, AI use in orthodontics, the belief that AI will lead to significant advancements in orthodontics, and the belief that AI will eventually replace orthodontists. Table 2 describes the characteristics of included studies in the present scoping review. Overall, 1772 total participants were identified across the studies. Among these, 1612 participants were considered eligible for the review. Of these, 1035 were orthodontists, including both academic and clinical practitioners, and 577 were orthodontic students or residents. The remaining 160 participants mentioned in the studies (e.g., dentists not specialized in orthodontics) did not fall within the predefined population of interest and were therefore excluded from subgroup analyses.

However, the included samples were heterogeneous in terms of population characteristics, including differences in professional experience, level of training, and exposure to AI technologies. Furthermore, the geographical distribution of the studies was limited and uneven, reflecting specific healthcare systems and educational contexts. This heterogeneity, together with the concentration of studies in certain regions, may influence the reported perceptions and limit the generalizability of the findings to the broader orthodontic community.

## 4. Discussion

This scoping review systematically mapped the existing literature on orthodontists’ perceptions of artificial intelligence, emphasizing overall attitudes, levels of knowledge, and concerns regarding its integration into orthodontic practice. Across the included studies, AI was generally regarded as a promising tool, particularly for diagnostic support and treatment planning, although variability in knowledge and acceptance was evident. As all studies utilized multi-section questionnaires, the discussion was structured according to the same thematic framework to enhance clarity and improve the readability of the findings.

However, the heterogeneity of the included samples, together with the relatively small number of participants and differences in professional experience, training background, and actual exposure to AI tools, limits the robustness of the available evidence. These factors make it difficult to extend the conclusions of this review to the broader population of orthodontists, highlighting the need for larger and more methodologically consistent studies.

### 4.1. AI Awareness

The reviewed studies showed that orthodontic professionals had varying degrees of awareness about AI, with academicians consistently demonstrating higher levels of knowledge compared to postgraduate students and clinicians. For instance, up to 25% of clinicians reported no awareness of AI, whereas 41.2% of academicians described themselves as extremely aware, compared to 20% of postgraduate students and 12.5% of clinicians [12]. Similarly, complete awareness of AI-based applications was reported by all academicians, compared to 75% of postgraduate students and only 50% of clinicians [12]. Although general awareness of the concept of AI was high (91.8%), substantial differences were observed in the depth of knowledge, particularly regarding advanced concepts such as ML and DL, which were more familiar to academicians than to clinicians [12].

Comparable findings were reported by Gupta et al. [15], confirming an awareness gap across professional roles, with faculty members showing greater familiarity than postgraduate students. Age and experience also appeared to influence perceptions, with senior professionals generally reporting fewer perceived barriers and slightly greater acceptance of AI, possibly due to cumulative exposure to technological advancements [20]. However, fewer than 75% of respondents across all groups reported more than moderate familiarity or confidence in using AI, indicating persistent uncertainty in its practical application [23].

Differences also emerged in sources of information: younger clinicians and students more frequently relied on web-based and social media sources, whereas academicians and senior professionals predominantly used formal academic channels such as lectures, conferences, and training programs [15,21,23]. Notably, undergraduate and postgraduate curricula contributed relatively little to AI knowledge acquisition, highlighting limited integration of AI education into formal training pathways [21]. Although 61.4% of respondents were aware of AI-based orthodontic software, 40.6% had never used such tools, suggesting a gap between theoretical awareness and clinical implementation [22]. Awareness was also uneven across specific applications, with high familiarity for cephalometric analysis but considerably lower awareness for areas such as orthognathic surgery planning and biomechanics [22].

However, these findings should be interpreted in light of the considerable heterogeneity of the included populations and study settings. Differences in geographic location, healthcare systems, educational structures, and access to technological resources may have significantly influenced both exposure to AI and reported levels of awareness. Additionally, differences by age, experience, gender, or academic seniority, variability in participants’ professional experience and training backgrounds further limit the comparability of results. As a consequence, the observed patterns may not be fully representative of the global orthodontic community, and caution is warranted when generalizing these findings.

Overall, the results suggest that professional role, experience, and access to educational resources influence awareness of AI, with academicians and more experienced practitioners generally demonstrating higher levels of familiarity. At the same time, the variability and fragmentation of knowledge across groups underscore the need for more structured and standardized AI education across all stages of orthodontic training [12,15,19,20,21,22,23].

### 4.2. Attitudes Toward AI

AI is generally viewed in modern orthodontic literature as a helpful clinical partner rather than a competitive replacement; however, the degree and type of this perception vary significantly depending on age, professional role, experience, and educational exposure. A large part (72%) of orthodontic academicians, clinicians, and postgraduate students specifically imagined AI as a partner in the foreseeable future of dentistry, particularly in diagnostic assistance and treatment planning, rather than autonomous decision-making. Moreover, a sizable portion (84%) of those viewed AI as a useful tool for improving professional performance and improving the quality of orthodontic care [12].

With 86% of respondents supporting AI’s role in orthognathic surgery planning and bone age assessment through CVMI staging and 90% thinking that AI-enhanced CBCT analysis represents a valuable diagnostic advancement for complex cases, there was a high degree of agreement regarding AI’s usefulness in managing complex clinical scenarios [12]. Although 75% of clinicians and 70.6% of academicians somewhat agreed that AI could be used to evaluate treatment outcomes, perceptions of AI as a quality control mechanism revealed significant professional differences. This statistically significant difference suggests greater practical confidence among clinicians directly involved in patient care [12].

In addition, 82% of postgraduates and only 59% of faculty members expressed willingness to use AI software in the future; future-oriented adoption intentions further highlight generational and hierarchical differences. Furthermore, postgraduates showed significantly stronger agreement overall, indicating higher receptivity among early-career orthodontists. Despite this openness, there was still some caution because there hasn’t been much agreement on incorporating AI into orthodontic treatment planning. This is because there aren’t many large-scale clinical trials or peer-reviewed studies that confirm AI’s safety and efficacy in actual clinical settings [15].

Educational expectations strongly reinforced the perception of AI as a collaborative tool, as 82% of postgraduates and approximately 72–73% of both postgraduates and faculty members advocated for AI to be an integral component of postgraduate training and future clinical practice following appropriate instruction, with younger participants expressing a significantly stronger desire to attend professional courses and hands-on training focused on AI technologies [15,19]. A proactive, learning-oriented approach to AI adoption rather than passive acceptance was highlighted by the consistent 74.2% of orthodontists who expressed a clear readiness to increase their knowledge of AI through lectures, workshops, and hands-on training sessions [19]. People with less than five years of experience and postgraduate status consistently showed significantly higher perception scores than those with over ten years of experience or faculty rank, indicating that younger professionals are more familiar with, confident in, and enthusiastic about AI technologies [15].

Conversely, attitude scores regarding the adoption of AI were paradoxically higher among those over 50, which may be due to the higher institutional authority and financial stability of professor-grade orthodontists. However, this group also exhibited lower levels of knowledge and more concern about AI interfering with clinical expertise [15]. Additionally, qualitative interpretations showed that younger orthodontists were more likely to trust AI as a complementary system because they had been exposed to recent technological advancements and had been immersed in digital environments, while older practitioners frequently expressed scepticism based on worries that machines could not replicate experiential clinical judgement [15].

This interpretive framework is further enhanced by gender-related differences. Female faculty members reported significantly higher perceptions of both opportunities and challenges associated with AI integration, suggesting heightened awareness of AI’s innovative potential alongside ethical concerns, training gaps, and implementation barriers. Contrary evidence suggests that sex-based disparities in AI readiness may vary across cultural contexts, particularly in Indian dental settings [20].

Due to the field’s heavy reliance on imaging, predictive modelling, and simulation technologies—where AI shows particular benefits in applications like cephalometric analysis, treatment simulation, and outcome prediction—orthodontists routinely showed higher opportunity scores and lower challenge perceptions than other dental specialists [20,21].

Despite evidence that such partnerships could mitigate instructor inadequacy and facilitate responsible AI integration, less than half of programs had established interdisciplinary collaborations with engineers, computer scientists, or AI companies, and 73.1% of programs did not require faculty teaching AI-related content to pursue continuing education [21,23]. While acknowledging time-saving potential and future relevance, the majority of orthodontists described AI’s impact on efficiency, accuracy, and patient experience as neutral. Only 3% of orthodontists consistently used AI, while 33.7% used it frequently, according to empirical perception data that also revealed cautious optimism in clinical settings [22].

However, these findings must be interpreted in light of the considerable heterogeneity of the included populations and the restricted geographical distribution of the studies. Variations in healthcare systems, educational frameworks, cultural contexts, and access to technological resources are likely to influence both attitudes and adoption patterns. Furthermore, differences in participants’ experience, training background, and exposure to AI tools reduce the comparability of results across studies. Overall, the evidence points to a largely positive attitude among orthodontists, who support AI as a complementary partner capable of improving diagnostic accuracy, treatment planning, and efficiency. At the same time, they emphasise the need for structured education, interdisciplinary collaboration, age- and gender-sensitive training strategies, and ethical governance frameworks to ensure that AI adoption supports clinical judgement and professional expertise rather than replaces it [12,15,19,20,21,22,23].

### 4.3. AI Recommendation

While attitudes toward AI were generally positive across all included studies, the extent to which orthodontists were willing to actively recommend its use or support its formal integration into clinical practice and education varied considerably. Overall, a majority of orthodontic academicians, clinicians, and postgraduate students (76%) supported recommending AI to peers, with only a small proportion expressing opposition, indicating broad professional acceptance [12]. However, this endorsement was consistently accompanied by the view that AI should function as a supportive tool rather than a replacement, with human clinical judgement retaining priority in decision-making, particularly in cases of disagreement between AI outputs and clinician expertise [12,24].

Recommendation patterns appeared to be influenced by generational and hierarchical factors. Postgraduate students demonstrated greater willingness to advocate for AI adoption compared to faculty members, suggesting higher receptivity among early-career professionals [15]. At the same time, a discrepancy emerged between individual attitudes and institutional practices. Data from North America indicated that a substantial proportion of orthodontic residency programs did not actively encourage the use of AI in clinical, research, or educational contexts, highlighting a gap between personal acceptance and organizational implementation [23]. This mismatch suggests the presence of structural and curricular barriers that may hinder the translation of positive attitudes into practice.

Educational integration represented the most consistently supported aspect, with the vast majority of respondents advocating for the inclusion of AI-related training in undergraduate and postgraduate curricula [22]. Despite this, evidence indicates that formal implementation remains limited in many programs, pointing to a lag between perceived importance and actual curricular adoption [15,22,23].

Conversely, these findings should be interpreted in light of the heterogeneity of the included populations and the geographical concentration of the studies. Differences in healthcare systems, institutional policies, educational frameworks, and access to technological resources are likely to influence both the willingness to recommend AI and the extent of its implementation. Moreover, variability in participants’ professional experience, training background, and exposure to AI tools reduces the comparability of results across studies. As a consequence, the observed trends may not be fully generalizable to the wider orthodontic community.

Overall, the evidence suggests a growing individual willingness—particularly among younger practitioners—to recommend AI and support its educational integration, contrasted by slower institutional adoption. This highlights the need for coordinated policy development, curriculum reform, and faculty engagement to align professional attitudes with practical implementation [12,15,22,23].

### 4.4. AI Application—Current Usage

According to the available data, AI is currently applied in orthodontics across multiple domains, including clinical practice, diagnosis, and treatment planning. Its use is primarily concentrated in diagnostic tasks such as cephalometric analysis, skeletal classification, facial scan interpretation, skeletal maturation assessment, upper airway evaluation, and CBCT analysis, as well as in treatment planning for extractions, orthognathic surgery, and outcome prediction. Additional applications include practice management, tele-orthodontics, and clinical documentation [12].

Despite high levels of perceived usefulness—such as strong support for AI integration in CBCT analysis (90%), orthognathic surgery planning (86%), and bone age assessment (84%)—a consistent gap emerges between perceived value and actual clinical use. For example, a substantial proportion of clinicians (62.5%) and postgraduate students (40%) reported not using AI for cephalometric analysis in daily practice [12]. Similarly, although most respondents acknowledged the potential of AI to improve patient communication and acceptance, its practical implementation remains limited [12]. Selective acceptance was also observed across applications, with higher agreement for diagnostic and imaging-related tasks than for AI-driven treatment planning, suggesting greater confidence in well-defined and image-based domains [15].

Institutional data further highlight the limited and often experimental nature of AI adoption. Only a proportion of postgraduate programs reported current or planned use of AI, most commonly in research, followed by diagnostic and monitoring applications, with less frequent use in treatment planning [23]. However, some areas—such as AI-assisted cephalometric analysis—show relatively higher levels of real-world adoption, driven by perceived advantages in time efficiency, accuracy, and usability, although many clinicians still rely on semi-automated rather than fully automated solutions [21,22]. Overall, usage patterns indicate a gradual transition in clinical workflows rather than a complete technological shift.

Nevertheless, these findings must be interpreted with caution due to the marked heterogeneity of the study populations and the geographical concentration of the available evidence. In summary, while AI is widely recognized as a valuable tool—particularly for image-based diagnostics and efficiency-driven tasks—its clinical application remains fragmented. Discrepancies between perceived potential, institutional readiness, and routine use underscore the persistence of a gap between technological capabilities and their consistent integration into orthodontic practice [12,15,21,22,23].

### 4.5. Concerns About AI

Concerns regarding the use of AI in orthodontics were multifaceted across the included studies, encompassing knowledge and training gaps, ethical and legal issues, accuracy and reliability, financial costs, and potential implications for professional roles. However, the relative importance of these concerns varied considerably across study populations and settings. A frequently reported barrier was the lack of technical knowledge and formal training, with higher levels of awareness significantly associated with prior exposure to AI in clinical or research contexts, suggesting that current limitations are primarily educational rather than attitudinal [15,21].

Financial aspects also emerged as a relevant concern, with a substantial proportion of respondents highlighting costs related not only to software acquisition but also to infrastructure and implementation, whereas data privacy concerns were less frequently reported [22]. Ethical considerations—including reduced human interaction, accountability in clinical decision-making, and the need for appropriate regulation—were also emphasized, alongside concerns about potential errors in AI-assisted treatment planning [12,15]. The absence of well-defined regulatory frameworks further complicates the integration of AI into healthcare, underscoring the importance of transparency, data protection, and robust validation processes. In particular, clinicians require greater insight into model development, data sources, and algorithmic decision-making to ensure trust and safe clinical application [25,26,27].

Concerns about professional replacement appeared relatively limited compared to other medical fields, with most respondents rejecting the notion that AI could fully substitute orthodontists, although some acknowledged its potential to outperform clinicians in specific diagnostic tasks [12,15,22]. Technical reliability remained a key issue, particularly in cephalometric analysis, where widespread manual correction of AI-generated outputs indicates persistent scepticism toward full automation [21]. This cautious approach is reinforced by variability in algorithm performance across datasets and development models, leading clinicians to prioritize their own judgement when discrepancies arise [21,22].

Educational and institutional barriers further contribute to these challenges, with limited curriculum time, lack of expertise, and insufficient interdisciplinary collaboration hindering effective AI integration [23,28]. Notably, younger orthodontists and postgraduate students tend to display greater openness and optimism toward AI adoption, whereas more experienced practitioners often express stronger concerns related to ethics, cost, and reliability, despite higher levels of familiarity [12,15,21,22,23].

However, these findings should be interpreted in light of the substantial heterogeneity of the included populations and the restricted geographical scope of the studies. Variations in healthcare systems, regulatory environments, educational structures, and access to technological resources are likely to influence both the type and intensity of concerns reported. Additionally, differences by age, experience, gender, or academic seniority, participants’ professional experience, training background, and exposure to AI tools further limit the comparability of results. As a consequence, the identified concerns may not be fully representative of the broader orthodontic community. Overall, these discrepancies highlight the need for context-specific strategies, including targeted education and practical training, to address barriers to AI adoption across diverse clinical and academic settings.

### 4.6. Limitations and Future Research

Several limitations of this review should be acknowledged. First, the available body of research on orthodontists’ perceptions of AI is limited in number, and the included studies exhibit substantial heterogeneity in terms of study populations, outcome measures, and methodological approaches, precluding any quantitative synthesis. In addition, the geographical distribution of the studies is relatively restricted, with most evidence originating from a limited number of countries, thereby reducing the external validity of the findings. Consequently, the results should be interpreted with caution, as they may not be fully generalizable to orthodontists practising in different regions or healthcare contexts.

Differences by age, experience, gender, or academic seniority, healthcare systems, educational frameworks, and access to digital technologies may significantly influence clinicians’ perceptions of AI. For instance, practitioners working in highly digitalized healthcare environments or within structured academic training programs that include AI-related content may demonstrate greater familiarity and more positive attitudes toward these technologies [23]. Conversely, limited access to technological resources or insufficient training opportunities may contribute to uncertainty, scepticism, or lower adoption rates.

Furthermore, all included studies relied exclusively on self-administered surveys with closed-ended questions, which may have constrained the depth and nuance of respondents’ perspectives. While this approach ensures standardized data collection and facilitates comparability across studies, it may not fully capture the complexity of orthodontists’ attitudes, concerns, and expectations regarding AI.

To gain a more comprehensive understanding of orthodontists’ perspectives, experiences, and reasoning related to AI adoption in clinical practice and education, future research should employ qualitative or mixed-methods designs. Approaches such as surveys with open-ended questions, semi-structured interviews, or focus groups would allow for richer insights into the underlying thought processes and contextual factors influencing AI integration in orthodontics.

## 5. Conclusions

In conclusion, this scoping review provides preliminary insights into orthodontists’ and orthodontic trainees’ perceptions of AI, suggesting a generally positive attitude and growing interest in its potential applications. However, these findings should be interpreted with caution, given that the evidence is limited to a low number of studies (restricted to cross-sectional survey data) and their heterogeneity in terms of design, populations, and outcomes. Considerable variability remains in levels of knowledge, current use, and perceived concerns, and the integration of AI into routine orthodontic practice appears inconsistent, potentially influenced by factors such as professional experience, role, and level of training.

Given the exploratory nature and restricted scope of the existing evidence, these results cannot be readily generalized across different settings. Nonetheless, they highlight the potential importance of institutional support, targeted educational strategies, and continued technological development in shaping future adoption. Efforts to bridge the gap between perceived potential and clinical implementation may benefit from the development of standardized guidelines, ethical frameworks, and structured educational pathways focused on AI.

Further research is warranted to strengthen the evidence base, particularly through studies employing more diverse methodologies, including qualitative approaches and open-ended data collection, to better capture the complexity of orthodontists’ perspectives. Ultimately, a cautious and evidence-based approach, supported by interdisciplinary collaboration, will be essential for the responsible integration of AI into orthodontic practice.

## Figures and Tables

**Table 1:** Terms used in the database search.

Database	Search Format
PUBMED	((“artificial intelligence”[Mesh] OR “artificial intelligence” OR “machine learning”[Mesh] OR “machine learning” OR “deep learning”) AND (“Orthodontists”[Mesh] OR orthodontist* OR “orthodontic resident*” OR “orthodontic student*” OR “dental student*”) AND (“Orthodontics”[Mesh] OR orthodontic* OR dental) AND (“Attitude to Computers”[Mesh] OR attitude* OR perception* OR view* OR opinion* OR belief* OR accept* OR adoption OR readiness OR confidence OR awareness OR survey* OR questionnaire*))
EMBASE via Ovid	(((‘artificial intelligence’ exp OR ‘machine learning’ exp OR ‘deep learning’ exp OR (artificial intelligence OR machine learning OR deep learning) ti,ab,kw) AND (‘orthodontist’ exp OR ‘orthodontics’ exp OR ‘dental student’ exp OR ‘dental education’ exp OR (orthodontist* OR orthodontic* OR “orthodontic resident*” OR “orthodontic student*” OR “dental student*”) ti,ab,kw) AND (‘attitude’ exp OR ‘perception’ exp OR ‘health attitude’ exp OR ‘technology acceptance’ exp OR (attitude* OR perception* OR view* OR opinion* OR belief* OR accept* OR adoption OR readiness OR confidence OR awareness OR survey* OR questionnaire*) ti,ab,kw))).af.
WEB OF SCIENCE	(“artificial intelligence” OR “machine learning” OR “deep learning”) AND (orthodontist* OR “orthodontic resident*” OR “orthodontic student*” OR “dental student*” OR dental OR “dental education”) AND (orthodontic* OR orthodontics OR dental) AND (attitude* OR perception* OR view* OR opinion* OR belief* OR accept* OR adoption OR readiness OR confidence OR awareness OR survey* OR questionnaire* OR knowledge*)
SCOPUS	(TITLE-ABS-KEY (“artificial intelligence” OR “machine learning” OR “deep learning”) AND TITLE-ABS-KEY (orthodontist* OR “orthodontic resident*” OR “orthodontic student*” OR “dental student*”) AND TITLE-ABS-KEY (orthodontic* OR orthodontics OR dental) AND TITLE-ABS-KEY (attitude* OR perception* OR view* OR opinion* OR belief* OR accept* OR adoption OR readiness OR confidence OR awareness OR survey* OR questionnaire*))

**Table 2:** Characteristics of the included articles in the scoping review.

Author/Year/Location	Sample	Type of Study	Objective	Conclusions
Al-Taie et al., 2025, Iraq [22]	101 orthodontists	cross-sectional study	To find out how specialised orthodontists view and feel about artificial intelligence in orthodontics.	Overall, specialists demonstrated a high level of readiness to participate in AI-related training and to integrate AI technologies into their clinical practice. A generally good level of awareness and knowledge regarding the role of artificial intelligence in orthodontics was also observed. However, to bridge the existing confidence gap, the study emphasizes the need for further training initiatives, enhanced educational programs, robust evidence-based validation, and the development of additional AI-driven solutions addressing multiple orthodontic domains, particularly biomechanics.
Ashwanthi et al., 2023, India [19]	360 members, out of which 191 orthodontists and 169 orthodontic residents	cross-sectional study	To use a questionnaire to evaluate orthodontists’ and orthodontic residents’ understanding, awareness, and application of digital orthodontics.	Despite limited experience in digital orthodontics, both orthodontic residents and practicing orthodontists demonstrated a positive attitude, an acceptable level of knowledge, and an overall favourable perception of emerging technologies.
Chutia et al., 2025, India [20]	250 dental faculty members, among which there are orthodontists (n = 90), prosthodontists (n = 70), pedodontists (n = 45) and periodontists (n = 45). Only the 90 orthodontists were included in the review analysis.	cross-sectional survey	Examining the potential of AI to improve learning systems and diagnostic tools, identifying obstacles including ethical issues and gaps in faculty training, and creating evidence-based adoption plans.	There was substantial faculty support for the integration of artificial intelligence, with perspectives influenced by professional role, specialization, and sex. To address existing barriers and enhance AI adoption within dental education, tailored training programs and curriculum revisions are warranted.
Gupta et al., 2025, India [15]	440 participants (264 postgraduates and 176 faculty members)	observational, cross-sectional survey	To assess postgraduate students’ and orthodontists’ knowledge, attitude, and perception (KAP) about the potential application of AI in orthodontics.	Most participants expressed optimism regarding the potential applications of artificial intelligence in orthodontics. However, despite a generally high level of awareness among orthodontists and postgraduate students, several barriers to the effective implementation of AI in clinical orthodontic practice were identified.
Hanenkrath et al., 2025, North America [23]	41 directors and program chairs	cross-sectional survey	To investigate the extent to which recognised postgraduate orthodontic programs in North America have incorporated the study and application of AI.	More than half of orthodontic residency programs have incorporated artificial intelligence into their curricula to some extent. Given the continuous advancements in AI algorithms, residency programs must adapt accordingly to remain aligned with technological progress. To equip residents with the competencies required for practice in an increasingly AI-driven clinical environment, structured AI-related education should be systematically integrated into the academic curriculum.
Lin et al., 2023, China [21]	480 participants, 298 orthodontists and 182 orthodontic students	cross-sectional survey	To evaluate orthodontists’ and orthodontic students’ knowledge, experience, and attitudes regarding AI-assisted cephalometric technologies; characterise their topic view of the applications and related technologies in orthodontics; and identify related factors.	Respondents expressed optimism regarding the future of artificial intelligence in orthodontics. AI-assisted cephalometric applications were widely perceived as likely to replace manual and semi-automated methods, thereby facilitating more efficient and convenient clinical diagnostic analysis. To enhance orthodontists’ understanding and promote informed adoption, greater emphasis should be placed on AI-focused education and awareness initiatives.
Mengi et al., 2024, Northern India [12]	100 participants, 50 orthodontists (academicians and clinicians) and 50 postgraduate students	Questionnaire study	To ascertain orthodontists’ knowledge, attitudes, and perceptions of artificial intelligence’s function in dentistry in general and orthodontics in particular, as well as how they use it.	Academicians demonstrated greater familiarity with artificial intelligence terminology and applications compared with postgraduate students and clinicians. There was a consensus that AI can support diagnostic procedures and treatment planning, thereby enhancing orthodontic performance and the quality of patient care. Nevertheless, despite these positive perceptions, 40% of postgraduate students and 62.5% of clinicians reported not using AI for cephalometric analysis.

## Data Availability

The original contributions presented in this study are included in the article and Supplementary Materials. Further inquiries can be directed to the corresponding author.

## Supplementary Materials

The following supporting information can be downloaded at: https://www.mdpi.com/article/10.3390/dj14050257/s1, File S1: PRISMA 2020 ScR checklist.

## Abbreviations

The following abbreviations are used in this manuscript:

AI	Artificial Intelligence
CBCT	Cone beam computed tomography
ML	Machine learning
DL	Deep learning
NLP	Natural language processing
PRISMA	Preferred Reporting Items for Systematic Reviews and meta-analyses
PRISMA-SCR	Preferred Reporting Items for Systematic Reviews and meta-analyses extension for scoping reviews
OSF	Open Science Frameworks
PCC	Population–Concept–Context
